# CNS Immune Profiling in a Dengue Virus-Infected Immunocompetent Outbred ICR Mice Strain

**DOI:** 10.3389/fcimb.2020.557610

**Published:** 2020-09-23

**Authors:** Ting-Jing Shen, Chia-Ling Chen, Ming-Kai Jhan, Po-Chun Tseng, Chiou-Feng Lin

**Affiliations:** ^1^Graduate Institute of Medical Sciences, College of Medicine, Taipei Medical University, Taipei, Taiwan; ^2^Department of Microbiology and Immunology, School of Medicine, College of Medicine, Taipei Medical University, Taipei, Taiwan; ^3^School of Respiratory Therapy, College of Medicine, Taipei Medical University, Taipei, Taiwan; ^4^Core Laboratory of Immune Monitoring, Office of Research & Development, Taipei Medical University, Taipei, Taiwan; ^5^Center of Infectious Diseases and Signaling Research, National Cheng Kung University, Tainan, Taiwan

**Keywords:** dengue virus, mice, CNS, cytokines, immune cells

## Abstract

Dengue virus (DENV) infection in the brain causes severe dengue disease with neuropathic complications. In addition to viral effects, immunogenic or pathogenic central nervous system (CNS) inflammation can be induced during DENV infection. By using an immunocompetent outbred ICR (Institute of Cancer Research) mouse model for investigating CNS immunity upon DENV infection, we conducted single-panel immune cell profiling and a multiplex cytokine assay. The ICR mice infected with DENV presented with progressive hunchback posture, limbic seizures, limbic weakness, paralysis, and lethality. When the virions were released, the viral non-structural protein 1 was expressed in the brain in a time-dependent manner. Isolated brain CD45-positive cells revealed a significant population of resident CD14-positive cells, which was considerably decreased 8 days post-infection. We found an unexpected time-kinetic decrease in CD19-positive cells and CD11c/MHC II-positive cells and an increase in NK1.1-positive cells. Further assays showed the time-dependent induction of proinflammatory and NK1.1-associated cytokines in the DENV-infected brains. These results indicate a CNS immune profile of DENV infection and hypothetical CNS immunity in response to DENV infection.

## Introduction

Dengue virus (DENV), a mosquito-borne *Flavivirus*, contains a positive-sense RNA genome that encodes three structural proteins (pr-M, envelope, and capsid proteins) and seven non-structural proteins (NS1, NS2A, NS2B, NS3, NS4A, NS4B, and NS5 proteins). The virus causes mild dengue fever (DF) and severe dengue diseases, including dengue hemorrhagic fever (DHF) and dengue shock syndrome (DSS), worldwide. Most DENV-infected people are asymptomatic or have mild symptoms, such as fever, headache, nausea, and joint pain. Unfortunately, some patients present with life-threatening warning signs, such as vascular leakage, internal bleeding, central nervous system (CNS) impairment, and multiorgan failure, that can result in death (Islam et al., 2015; Guzman et al., 2016; Ajlan et al., 2019).

Many studies have shown that DENV can infect various human organs (Shresta et al., 2004; Povoa et al., 2014; Milligan et al., 2015). The DENV negative-sense RNA and viral protein NS3 are detectable in the hearts of the deceased (Shresta et al., 2004). In addition, DENV positive-sense RNA and another viral antigen, NS1, can also be detected in human spleen, lung, liver, and CNS tissues (Povoa et al., 2014). Upon DENV infection, AG129 mice show a widespread distribution of infectious viruses in multiple organs, including their livers, spleens, and large intestines, as well as their brains and spinal cords (Shresta et al., 2004; Milligan et al., 2015). Sarathy et al. also demonstrated the dynamic changes in viral loads in DENV 3-infected AG129 mice. High levels of viral titers were found in livers and spleens starting on the first day of infection. The productive virions had increased in the large intestines and brains at 2 and 3 days post-infection (d.p.i.), respectively. Lethal dengue disease is characterized by the robust induction of cytokines and chemokines in mouse serum, such as interleukin (IL)-6, IL-10, and interferon (IFN)-γ, showing a high correlation with infectious disease progression (Sarathy et al., 2015).

In DENV-infected patients, aberrant production of proinflammatory cytokines and chemokines, such as tumor necrosis factor (TNF)-α, IFN-γ, granulocyte-macrophage colony-stimulating factor (GM-CSF), IL-6, IL-8, IL-10, and C-X-C motif chemokine 10 (CXCL-10), which is the critical marker of disease severity. Hypothetically, endothelial cells, monocytes, macrophages, dendritic cells (DCs), natural killer (NK) cells, and T cells are thought to produce these soluble inflammatory factors in DENV-infected patients and mice (Durbin et al., 2008; Costa et al., 2013; Schmid and Harris, 2014; Singla et al., 2016; Patro et al., 2019). Many studies have indicated that DENV can infect the neural system and induce neurological symptoms in humans and mice (Amaral et al., 2011; Verma et al., 2014); however, no reports have shown a CNS inflammation profile for DENV infection. The goals for a systemic study to monitor the kinetic changes of DENV virions and host immune responses have therefore been unmet.

In this study, as shown through the use of an immunocompetent ICR suckling mouse model similar to that created previously (Shen et al., 2019), DENV infection induced severe disease in the mouse CNS, which caused neural symptoms. An immune profiling analysis of the brain revealed dynamic changes in the immune cells and inflammatory responses.

## Materials and Methods

### Ethics Statement

The Ethics Committee approved all experimental procedures used in the animal work on the Institutional Animal Care and User Committee of National Defense Medical Center, Taipei, Taiwan, protocol IACUC 16-261.

### Cells and Virus

Dengue virus serotype 2 (DENV2, strain PL046) was obtained from Center Disease Control in Taiwan. We propagated in the monolayer of *Aedes albopictus* clone mosquito C6/36 cells (ATCC, CRL1660) at a multiplicity of infection (MOI) of 0.01 and incubated the cells at 28°C in 5% CO_2_ for 5 days. The viral supernatants were collected and filtered with a 0.22 μm filter and then stored at −80°C until use. The C6/36 cells were maintained in minimum essential medium (MEM; Thermo Fisher Scientific) containing 10% heat-inactivated fetal bovine serum (FBS, Biological Industries), 1% penicillin-streptomycin (Thermo Fisher Scientific), 1% sodium pyruvate, 1% 4-(2-hydroxyethyl)-1-piperazine ethanesulfonic acid (HEPES; Thermo Fisher Scientific), and 1% non-essential amino acids (NEAA; Thermo Fisher Scientific) at 28°C in 5% CO_2_. Baby hamster kidney (BHK)-21 fibroblasts (ATCC, CCL10) were cultured in Dulbecco's modified Eagle's medium (DMEM; Thermo Fisher Scientific), 10% heat-inactivated FBS, and 1% penicillin-streptomycin at 37°C in 5% CO_2_.

### Infectious Animal Model

To establish the animal model as in previous studies (Tsai et al., 2016; Ho et al., 2017; Jhan et al., 2018; Kao et al., 2018; Shen et al., 2019), we inoculated 7-d-old ICR suckling mice (BioLASCO, Taiwan) with 2.5 × 10^5^ and 7.5 × 10^5^ plaque-forming units (pfu) of DENV 2 (PL046) by simultaneous intracerebral and intraperitoneal injections, respectively. The survival rate and disease score of the mice were monitored daily and recorded. The following disease scoring guidelines were used: 0 for healthy mice; 1 for mice with minor symptoms of illness, including weight loss, reduced mobility, and a hunchback body orientation; 2 for mice that exhibited limbic seizures; 3 for mice that showed limbic weakness, including difficulty moving and anterior or posterior limb weakness; 4 for paralysis; and 5 for death.

### Antibodies

Antibody against DENV NS1 (Cat#GTX124280) was purchased from GeneTex (San Antonio, TX); antibody against mouse β-actin (Cat#A5441) was purchased from Sigma-Aldrich (St. Louis, MO); horseradish peroxidase (HRP)-conjugated goat anti-rabbit IgG (Cat# 7074S), HRP-conjugated horse anti-mouse IgG (Cat# 7076S) were purchased from Cell Signaling Technology (Beverly, MA); antibodies against mouse CD45 (Cat# 56-0451-82), CD3 (Cat# 48-0031-82), CD11c (Cat# 12-0114-82), CD14 (Cat# 17-0141-82), CD19 (Cat# 69-0193-82), MHC class II (Cat#67-5321-82), and NK1.1 (Cat# 69-5941-82) were purchased from Thermo Fisher Scientific.

### Western Blotting

The murine organs were homogenized and extracted with lysis buffer containing a protease inhibitor mixture (Sigma-Aldrich). The extracted proteins were quantified to be of equal concentration and mixed with protein dye. Then, the processed proteins were separated by SDS polyacrylamide gel electrophoresis for 2 h, followed by transfer to a polyvinylidene difluoride (PVDF) membrane (Millipore). The PVDF membrane was blocked with 5% non-fat milk in Tris-buffer-based saline containing 0.05% Tween-20 (TBS-T) at room temperature for 1 h. The membrane was washed three times with TBS-T buffer. Next, the membrane was immunohybridized with the indicated primary antibodies at 4°C for 16–18 h. Then, the membrane was washed with TBS-T buffer three times and then incubated with the indicated HRP-conjugated secondary antibodies. PVDF membranes containing antibody-protein complexes were detected using an ECL Western blot detection kit (PerkinElmer). Then, the relative densities of the identified proteins were quantified using ImageJ software (Fiji Software).

### Plaque Assay

BHK-21 cells were seeded in a 12-well plate (7 × 10^4^ cells/ml) and allowed to form a monolayer overnight. Samples were diluted at 10^−1^-10^−5^ and then incubated with BHK-21 cells at 37°C in 5% CO_2_ for 2 h. Next, the virus inoculum was discarded and replaced with DMEM containing 4% FBS and 0.5% methylcellulose (Sigma-Aldrich) for 5 days. The wells containing methylcellulose were washed with 2 ml PBS twice and then fixed and stained overnight with a crystal violet solution containing 1% crystal violet (Sigma-Aldrich), 0.64% NaCl, and 2% paraformaldehyde (Sigma-Aldrich). The stain was removed with a water wash, and the plates were dried at room temperature. Viral plaques were counted by visual observation.

### Multiplex Assay

Mouse brains were homogenized with 400 μl of iced-PBS on ice, followed by centrifugation at 12,000 rpm for 30 min. Supernatants were harvested and quantified to an equal concentration of 1 μg/μl in at least 35 μl. The assay was performed by using an Immunology Multiplex Assay MTH17MAG-47K (Millipore) according to the manufacturer's instructions. Briefly, 200 μl of wash buffer was added to each well of the 96-well plate and shaken at room temperature for 10 min, and then, the wash buffer was removed. Then, 25 μl of standard/control and 25 μl of assay buffer were added to the indicated wells and the background/sample wells, respectively. Next, 25 μl of an appropriate matrix solution was added to the standards, controls, and background wells. Twenty-five microliters of each sample and 25 μl of antibody-immobilized beads were added to the wells. After overnight incubation at 4°C, the supernatant was gently removed, and the plate was washed twice with 200 μl of wash buffer. Then, 25 μl of detection antibody was added to each well, and the plate was incubated at room temperature. After a 1-h incubation, 25 μl of streptavidin-phycoerythrin was added to each well and incubated at room temperature for 30 min. The supernatant was gently removed, and the plate was washed twice with 200 μl of wash buffer. Then, 150 μl of sheath fluid was added per well. After incubation of the plate on a shaker for 10 min, the reactions were detected and analyzed by using Luminex MAGPIX® with xPONENT® software.

### Flow Cytometry

Mouse brains were homogenized with 5 ml of iced wash buffer (HBSS + 10% FBS) and then centrifuged at 400 × g for 5 min at 4°C. The supernatant was discarded, and the pellet was resuspended in 1 ml of digestion buffer (ACCUTASE® solution). The samples in the digestion buffer were gently rotated at room temperature. After a 1-h incubation, the samples were washed with 1 ml of wash buffer and centrifuged at 400 × g for 5 min at 4°C. Then, the samples were gently mixed with 5 ml of 25% density gradient medium (nine parts of Percoll with one part of 10 × HBSS as a 100% isotonic density gradient stock medium, which was then further diluted with HBSS containing 3% FBS) and centrifuged at 600 × g for 20 min at 4°C. The myelin coat and the supernatant were carefully removed. One milliliter of wash buffer was added to the cell pellet, and the samples were centrifuged at 400 × g for 5 min at 4°C. After washing the brain samples, 1 ml of staining buffer was added, and the cells were counted. The samples were immunoblocked (Mouse BD Fc Block™) at 4°C. After 30 min, the cells were immunohybridized with specific antibodies against cell surface immune markers, including CD45, CD3, CD11c, MHC class II, CD14, CD19, and NK1.1, at 4°C for 1 h. Then, the cells were washed with 500 μl of iced staining buffer and resuspended in 500 μl of iced staining buffer. The samples were analyzed using a flow cytometry (Attune Nxt) system.

### Statistical Analysis

Experimental data were analyzed using GraphPad Prism (Version 8.3.0). Unpaired *t*-test and one-way ANOVA (Tukey's multiple comparisons test) were used to determine experiments involving two and various groups, respectively. The survival rate followed a log-rank test. Values are means ± standard deviation (SD). All *p*-values are for two-tailed significance tests. A *p*-value of < 0.05 is considered statistically significant.

## Results

### DENV Infection Induces CNS Impairment and Death in ICR Suckling Mice

**Figure 1:**
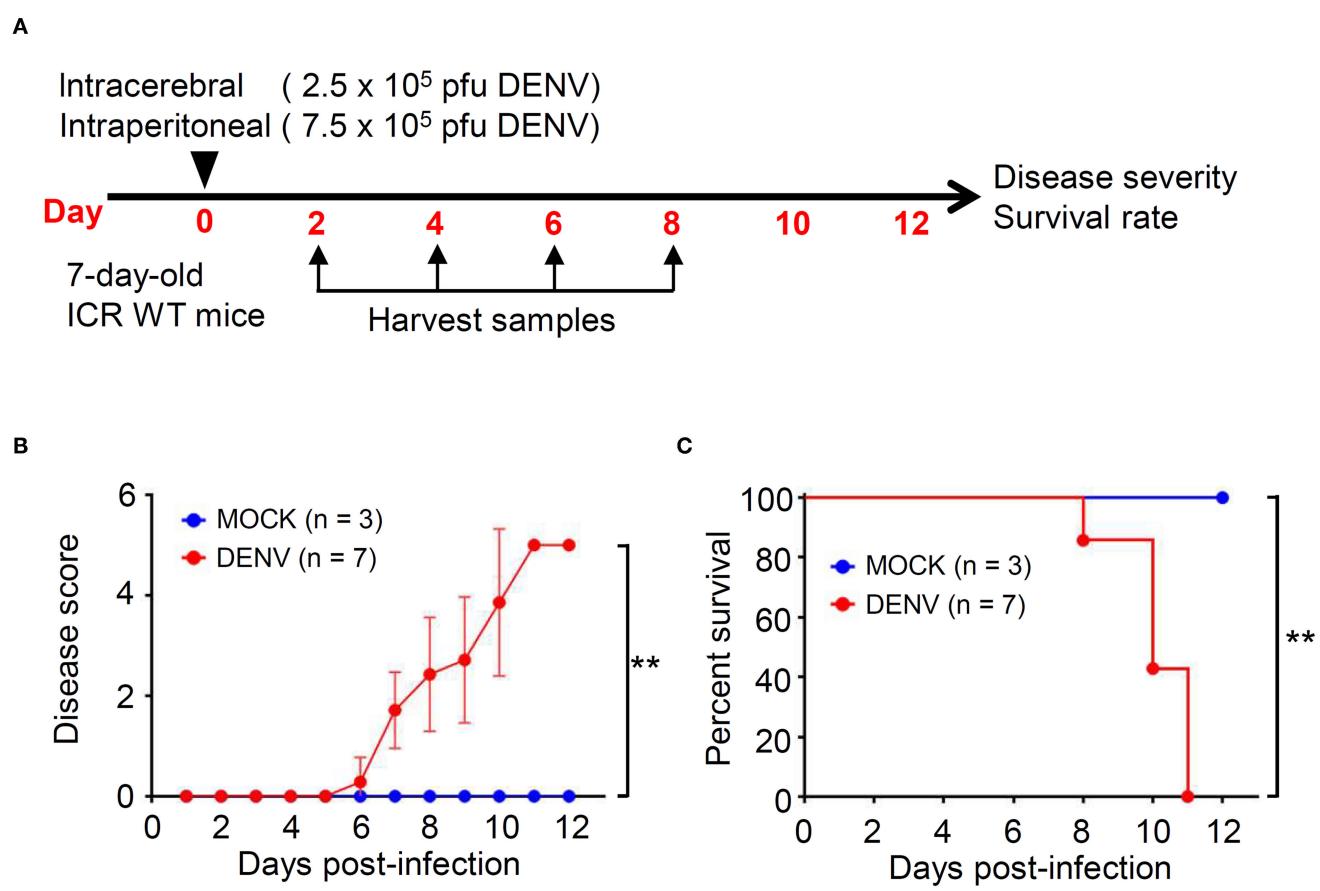
DENV infection causes symptoms and death in ICR immunocompetent mice. **(A)** A concurrent intracerebral and intraperitoneal injection model of DENV infection was generated in 7-days-old ICR mice. Organs/tissues were harvested at the indicated day post-infection. We monitored **(B)** the disease score and **(C)** the survival rate of mice for 12 days after infection. Wilcoxon signed-rank test and log-rank test analyzed the disease score and the survival rate, respectively. The values are presented as means ± SD. **p < 0.01.

We established a DENV infection model in immunocompetent mice, according to our previous study (Shen et al., 2019). Seven-days-old ICR suckling mice were simultaneously infected with 2.5 × 10^5^ and 7.5 × 10^5^ PFU/ml of DENV by intracerebral and intraperitoneal injections, respectively (Figure 1A). Compared with that shown by the mock group, the DENV-infected mice showed significantly increased disease severity with symptoms (*p* < 0.01), including reduced mobility, limbic seizure, and paralysis within 6 d.p.i. (Figure 1B). Moreover, the survival rates were significantly different, with the DENV-infected mice dying after 8 d.p.i., and all mice dying within 11 d.p.i. (*p* < 0.01) (Figure 1C). The results reveal the establishment of a reporter DENV-infected murine model with disease progression involving CNS disorders.

### DENV Significantly Causes Infection in the Mouse CNS

**Figure 2:**
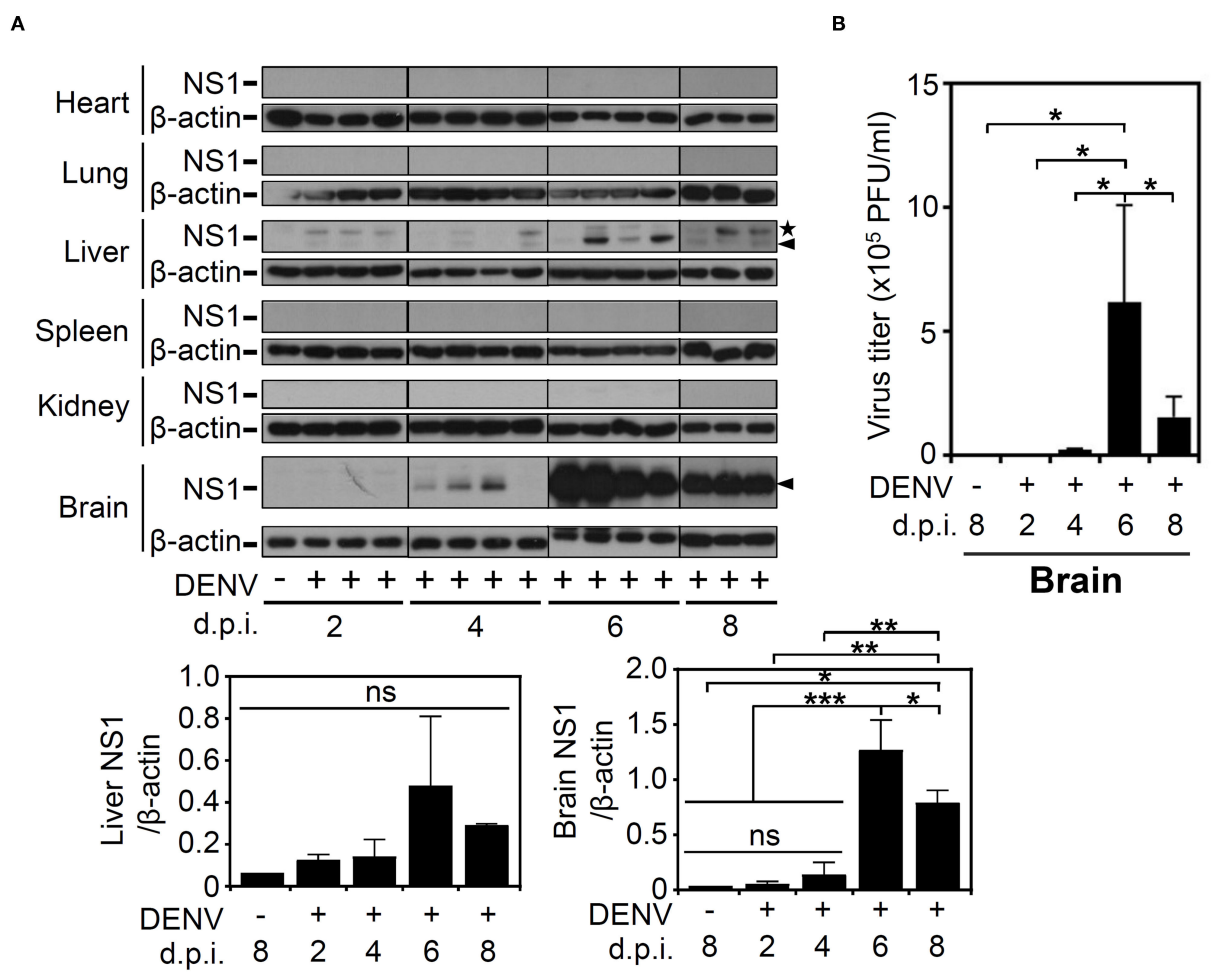
DENV infection causes NS1 protein expression in multiple organs, especially induces virion production in mouse brains. **(A)** Western blot analysis showed viral NS1 protein expression in various organs of the mice. β-actin was used as internal control. Arrowhead (▴): NS1 protein. Star (**⋆**): non-specific protein. **(B)** The plaque assay determined the viral titer in the infected brains. Data are presented as the means ± SD based on at least three mice. *p < 0.05. Data are presented as the means ± SD based on at least three mice. *p < 0.05; **p < 0.01; ***p < 0.001. ns: not significant.

Based on the successful infection of this murine model, we next evaluated the infectious target of DENV. We harvested various organs/tissues for viral protein detection using Western blot analysis. According to the results, there was detectable viral NS1 protein in the livers of the mice post-infection but not in the other organs, including the hearts, lungs, spleens, and kidneys. However, in the mouse brains, NS1 was significantly (*p* < 0.001) expressed at 6 d.p.i., and the expression was significantly (*p* < 0.05) decreased at 8 d.p.i. (Figure 2A). To confirm the infectivity and viral replication in the murine CNS, a plaque assay was conducted, and the results demonstrated a titer of productive virions in the brain representing a remarkable increase at 6 d.p.i. (*p* < 0.05) and a decrease at 8 d.p.i. (*p* < 0.05), findings that closely matched the NS1 expression results (Figure 2B). In addition, although the NS1 protein was recognized, the results of the plaque assay showed that there was no detectable virion in mouse liver (data not shown). The data show that DENV could infect the CNS and effectively replicate it.

### DENV Infection Causes Changes in the Immune Cell Profile of the Mouse Brain

**Figure 3:**
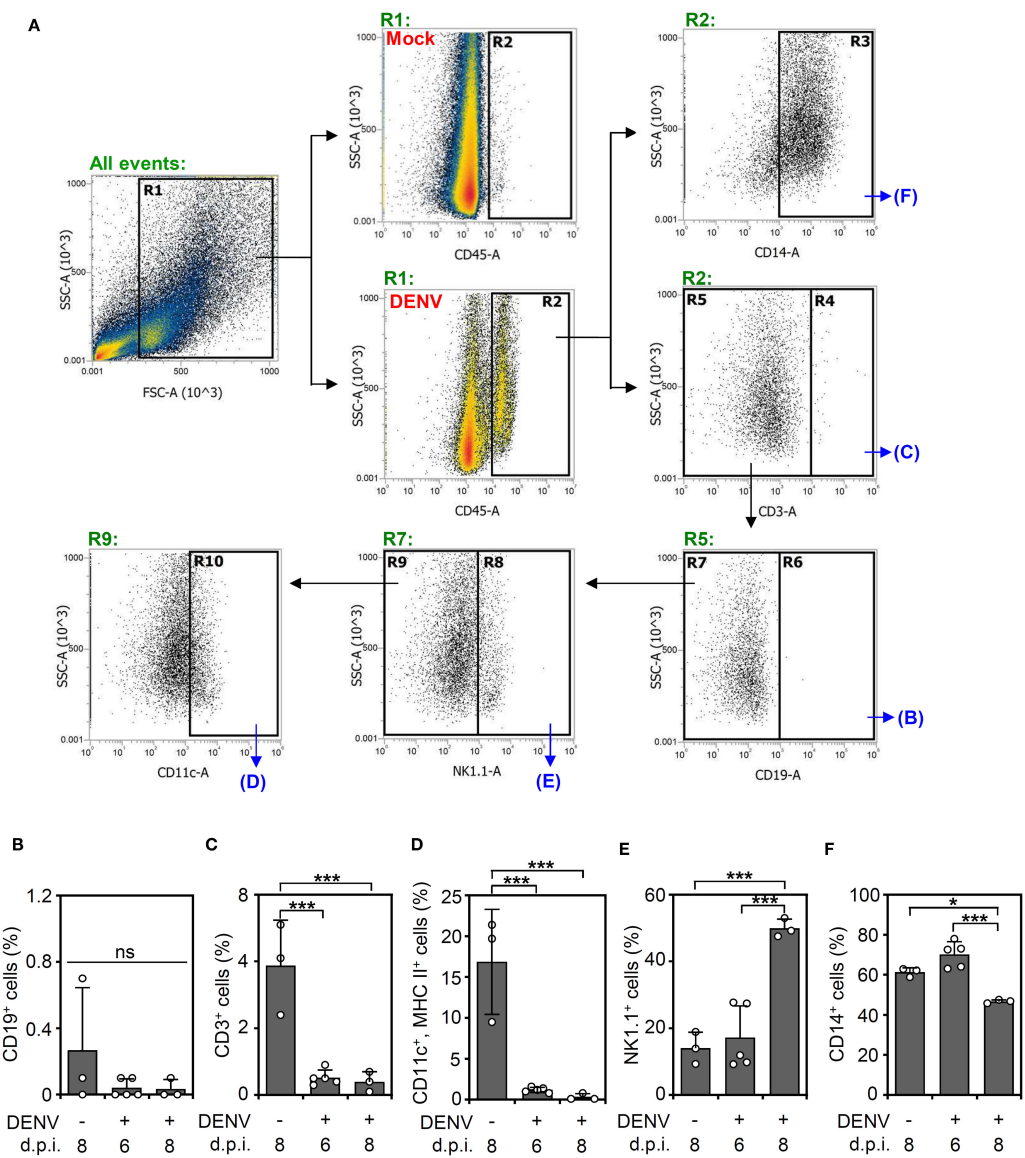
DENV infection causes dynamic changes in immune cell populations in the brain. **(A)** A gating immunostaining strategy was used for the indicated cell populations. The percentage of **(B)** CD19-positive cells, **(C)** CD3-positive cells, **(D)** CD11c and MHC II-positive cells, **(E)** NK1.1-positive cells, and **(F)** CD14-positive cells in the immune cell populations of the mouse brains on the indicated day post-infection were shown. Each point represents a mouse sample. Data show the means ± SD of based on at least three mice. *p < 0.05; ***p < 0.001. ns: not significant.

According to the levels of viral proteins and viral titers (Figure 2), we created a successful DENV infection in the CNS of immunocompetent mice. To further investigate using single-shot immune cell profiling of the CNS in the mice, immunostaining of a panel of CD45, CD3, CD11c, MHC class II, CD14, CD19, and NK1.1 was performed in DENV-infected brain cells isolated from suspension. A gating strategy was applied to the CD45-positive cells to distinguish the indicated cell populations (Figure 3A). According to the results, the percentage of CD19-positive cells (Figure 3B) remained relatively unchanged. The percentage of CD3-positive cells (Figure 3C) and CD11c and MHC class II-positive cells (Figure 3D) was significantly (*p* < 0.05) decreased, while that of the NK1.1-positive cells (Figure 3E) was significantly (*p* < 0.001) increased within days of DENV infection. The significant population consisted of resident CD14-positive cells (Figure 3F), likely microglia, in the DENV-infected mouse brains, which increased at 6 d.p.i. but remarkably decreased at 8 days post-infection. Collectively, the results demonstrate that DENV infection orchestrates the composition of immune cells in the CNS of immunocompetent mice.

### DENV Infection Induces Cytokine and Chemokine Storms in the Mouse CNS

**Figure 4:**
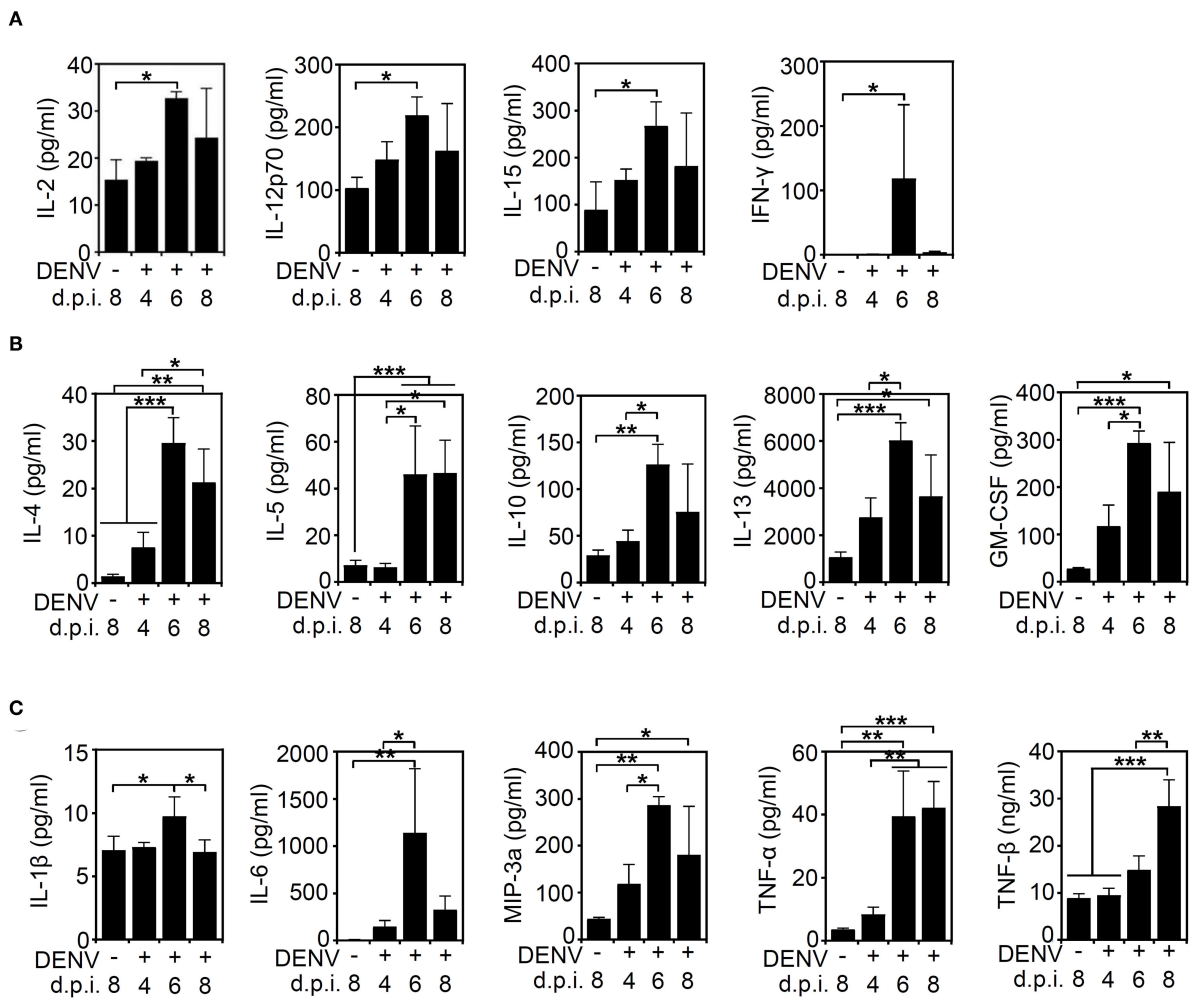
DENV infection induces cytokine/chemokine production in the brain. A multiplex assay showed the levels of **(A)** type 1-like cytokine/chemokines (IL-2, IL12p70, IL-15, and IFNγ), **(B)** type 2-like cytokine/chemokines (IL-4, IL-5, IL-10, IL-13, and GM-CSF), and **(C)** proinflammatory proteins (IL-1β, IL-6, MIP-3a/CCL20, TNF-α, and TNF-β) produced in the brains of mice on the indicated day post-infection. Data show the means ± SD based on at least three mice. *p < 0.05; **p < 0.01; ***p < 0.001. ns, not significant.

After we found that high viral protein and viral loads were associated with neurological and physiological changes in the infected mice, we determined the immune profile of the mouse CNS. Because of the increased expression of NK1.1-positive NK cells in the DENV-infected murine brain, the levels of other immune parameters needed to be validated. Elevated levels of cytokines and chemokines, including IL-10, IFN-γ, TNF-α, GM-CSF, and MIP-1β, are potential biomarkers of disease severity in DENV patients (Patro et al., 2019). In consideration of an increased NK cell response, the expression of cytokines/chemokines associated with NK cell activation was hypothetically measured according to methods used in previous works (Peritt et al., 1998; Biron et al., 1999; Cooper et al., 2001; Fauriat et al., 2010). The multiplex assay was performed to measure the production of cytokines/chemokines in the DENV-infected mouse brains. Compared to those of the mock group, the levels of cytokines and chemokines, including NK-associated type 1 cytokines (IL-2, IL12p70, IL-15, and IFN-γ) (Figure 4A), type 2 cytokines (IL-4, IL-5, IL-10, IL-13, and GM-CSF) (Figure 4B), and proinflammatory factors (IL-1β, IL-6, MIP-3a/CCL20, TNF-α, and TNF-β) (Figure 4C), were significantly increased (*p* < 0.05) within days of induced DENV infection. These results demonstrate that DENV infection can induce the overproduction of inflammatory cytokines/chemokines in the brain.

## Discussion

An animal model of DENV infection is crucial for exploring the pathogenesis of dengue diseases and for evaluating the antiviral strategies, particularly vaccine development (An et al., 1999; Guabiraba and Ryffel, 2014; Sarathy et al., 2015, 2018). Despite the limitations on immunocompromised mice, when monitoring the immune response is necessary, an immunocompetent murine model is essential. We created a murine model of DENV infection in immunocompetent mice (Tsai et al., 2016; Shen et al., 2019). Through a time-kinetic analysis, this work not only demonstrated a significant replication of DENV in the CNS but also identified the possible immune parameters, including immune cells and cytokines/chemokines, related to viral replication *in vivo*. The increase in NK1.1-positive cells and their regulatory cytokines and chemokines was demonstrated in this infectious model, and it was closely associated with viral load. Immune profiling of the DENV-infected brain may provide findings showing possible viral immunity in the CNS following DENV infection.

Innate immune responses, such as the induction of type 1 and 2 IFNs, are crucial for the host defense against DENV infection, while IFN-triggered antiviral responses reduce viral replication and viral dissemination (Liang et al., 2011; Suzuki et al., 2016; Balinsky et al., 2017; Sprokholt et al., 2017). Studies widely use immunocompromised AG129 mice, which lack IFN-α/β/γ receptors, to investigate DENV infection. In these mice, mosquito bites lead to natural DENV infection in various organs/tissues, including the lung, liver, spleen, intestine, and brain (Shresta et al., 2004; Milligan et al., 2015; Sarathy et al., 2015, 2018). However, the results of the current study showed a mild infection in the liver and a severe infection in the brain of infected mice. Through an intravenous route of infection using immunocompetent C57BL/6 mice, Chen et al. found that dengue viral RNA was detectable in the spleen, liver, and brain. Additionally, the production of IFN-γ and the levels of helper and cytotoxic T cells have been found in the spleens of infected mice (Chen et al., 2004). Immunocompetent mice present disorders similar to those shown in the clinic, including viral hepatitis and neuropathy, by patients with severe dengue disease (Povoa et al., 2014; Verma et al., 2014). Further pathological studies related to CNS immunity are required to support the application of the present model of DENV infection, particularly concerning the liver and CNS.

Notably, CNS symptoms have been identified as critical signs of severe DENV disease since 2009 (WHO, 2009). Severe dengue diseases include the involvement of neuropathy and viral encephalitis (Carod-Artal et al., 2013; Verma et al., 2014). The construction animal models of DENV infection with neural complications are urgently needed for further investigation. According to our current work, which is similar to that of previous works using immunocompetent BALB/c mice (Amorim et al., 2019) or C57BL/6 mice (Chen et al., 2004), viral RNA was significantly increased in the brain 1 week after DENV was inoculated through intravenous and intracerebral routes of infection. These infection models demonstrated the successful viral infection and replication in the CNS, indicating a viral enhancement in immunocompetent mice under precise immune-privileged conditions. As noted in the mice that died from viral encephalitis-like symptoms, neurotoxicity caused by viruses may be directly and indirectly affected by excessive CNS inflammation (Verma et al., 2014). Additionally, our previous works demonstrated that activated microglia act as antigen-presenting cells (APCs) for controlling type 1 antiviral immune responses (Tsai et al., 2016). CNS immunity may be pathogenic under neurotoxic conditions and protective in antiviral responses.

According to the immune cell profiling results, we showed that CD45^+^CD14^+^ cells were the major immune cell components in the brains of the naïve mice, while there was a significant decrease in this cell population at 8 d.p.i. DENV infection. Microglia, immune cell resident in the brain, express CD14 as an immune organizer in response to CNS infection and inflammation (Janova et al., 2016). However, under IFN-γ and IL-14 stimulation, the expression of CD14 is significantly decreased by an extremely flexible regulation response. The suppression of CD14 is speculated to be required to weaken the damage response and to favor a strong defense in response to infection. Additionally, activated macrophages, which express downregulated CD14, are involved in macrophage polarization (Genin et al., 2015). Our previous studies and those of others also demonstrated the induction of microglia-derived APCs and the presence of macrophages during antiviral activity (Fink et al., 2009; Tsai et al., 2016). The decreased CD14-positive cells may indicate flexible changes in CNS immunity and require further investigation.

Transcriptomic analysis of dengue patient blood showed a positive correlation of monocytes, macrophages, DCs, and neutrophils, and a negative correlation of NK cells, CD4^+^ T cells, CD8^+^ T cells, and B cells with viral loads (Kwissa et al., 2014). Our results also showed late production of NK1.1-positive cells that ultimately constituted a relatively high percentage of the immune cell populations in the brains of the DENV-infected mice, while the viral load was relatively diminished. In general, NK cells play diverse roles in defending against infection (Freud et al., 2017). Many Dengue studies have demonstrated that antiviral NK cells cooperate with IFNs and TNF-α to eliminate virus from DENV-infected DCs (Lim et al., 2014) and DENV-infected mice (Costa et al., 2017). However, infiltrating NK cells have been reported to cause liver cell death (Sung et al., 2012). Therefore, more exploration is needed to examine the critical roles of immune cells, such as NK1.1-positive cells and CD14-positive monocytes/macrophages/microglia, during DENV infection, especially in the CNS.

Generally, TNF-α, as well as IL-4, IL-6, IL-8, and IL-10, are increased in dengue patients (Butthep et al., 2012). We previously demonstrated that DENV infection causes overt TNF-α production during the progression of viral encephalitis (Jhan et al., 2018). The cross-talk between aberrant cytokines/chemokines and immune cells in the CNS needs to be explored. To validate the induction of high NK1.1-positive cell levels in the brains of DENV-infected mice, we analyzed the elevated production of cytokines/chemokines that are highly correlated with intensive NK cell infiltration (Peritt et al., 1998; Biron et al., 1999; Cooper et al., 2001; Fauriat et al., 2010). Our results showed that all NK-associated type 1 cytokines (IL-2, IL12p70, IL-15, and IFNγ), type 2 cytokines (IL-4, IL-5, IL-10, IL-13, and GM-CSF), and proinflammatory factors (IL-1β, IL-6, MIP-3a/CCL20, TNF-α, and TNF-β) were increased within 6 d.p.i. of DENV infection while the level of NK cells was significantly increased at 8 d.p.i. The role of NK1.1-positive cells was undefined in this work, but IL12p70 and IFNγ may have induced the NK cell differentiation toward the type 1 form, and the type 2 form may have been induced by IL-4 (Peritt et al., 1998; Kimura and Nakayama, 2005).

In conclusion, our findings provide a possible link among CNS immunity, viral tropism, and host immune responses during DENV infection in the brain. The induction of dynamic cytokine/chemokine response and changes in immune cell profiling may indicate a link between viral load and CNS impairment and/or protection for CNS immunity. For future studies, particularly for evaluating the efficacy of the vaccine and immune therapy, solving the whole puzzle of viral pathogenesis and host responses in CNS upon DENV infection must be completed by comparing different sources of virus, infectious routes, viral strains, viral inocula, and animal models.

## Data Availability Statement

The datasets generated and analyzed for this study are available from the corresponding author upon request.

## Ethics Statement

The animal study was reviewed and approved by the Institutional Animal Care and User Committee of National Defense Medical Center, Taipei, Taiwan, protocol IACUC 16-261.

## Author Contributions

T-JS performed most of the experiments and interpreted the results. C-FL and C-LC participated in the design and supervision of the projects. T-JS and M-KJ conducted the mouse experiments. P-CT contributed to the flow cytometry analysis. T-JS and C-FL designed the concept of the project and wrote the manuscript. All authors reviewed and approved the manuscript.

## Conflict of Interest

The authors declare that the research was conducted in the absence of any commercial or financial relationships that could be construed as a potential conflict of interest.
